# ROS-Mediated Apoptotic Cell Death of Human Colon Cancer LoVo Cells by Milk δ-Valerobetaine

**DOI:** 10.1038/s41598-020-65865-6

**Published:** 2020-06-02

**Authors:** Nunzia D’Onofrio, Nunzio Antonio Cacciola, Elisa Martino, Francesca Borrelli, Ferdinando Fiorino, Assunta Lombardi, Gianluca Neglia, Maria Luisa Balestrieri, Giuseppe Campanile

**Affiliations:** 1 Department of Precision Medicine, University of Campania L. Vanvitelli, 80138 Naples, Italy; 2 Department of Veterinary Medicine and Animal Productions, University of Naples Federico II, 80137 Naples, Italy; 3 Institute of Sustainable Plant Protection (IPSP), National Research Council (CNR), 80055 Naples, Italy; 4 Department of Pharmacy, School of Medicine and Surgery, University of Naples Federico II, 80131 Naples, Italy; 5 Department of Biology, University of Naples Federico II, 80126 Naples, Italy

**Keywords:** Biochemistry, Cancer, Cell biology, Molecular biology

## Abstract

δ-Valerobetaine (δVB) is a constitutive milk metabolite with antioxidant and anti-inflammatory activities. Here, we tested the antineoplastic properties of milk δVB on human colorectal cancer cells. CCD 841 CoN (non-tumorigenic), HT-29 (p53 mutant adenocarcinoma) and LoVo (APC/RAS mutant adenocarcinoma) cells were exposed to 3 kDa milk extract, δVB (2 mM) or milk+δVB up to 72 h. Results showed a time- and dose-dependent capability of δVB to inhibit cancer cell viability, with higher potency in LoVo cells. Treatment with milk+δVB arrested cell cycle in G2/M and SubG1 phases by upregulating p21, cyclin A, cyclin B1 and p53 protein expressions. Noteworthy, δVB also increased necrosis (*P* < 0.01) and when used in combination with milk it improved its activity on live cell reduction (*P* < 0.05) and necrosis (*P* < 0.05). δVB-enriched milk activated caspase 3, caspase 9, Bax/Bcl-2 apoptotic pathway and reactive oxygen species (ROS) production, whereas no effects on ROS generation were observed in CCD 841 CoN cells. The altered redox homeostasis induced by milk+δVB was accompanied by upregulation of sirtuin 6 (SIRT6). *SIRT6* silencing by small interfering RNA blocked autophagy and apoptosis activated by milk+δVB, unveiling the role of this sirtuin in the ROS-mediated apoptotic LoVo cell death.

## Introduction

Milk health benefits reflect its abundance in bioactive peptides with antimicrobial, anticancer, immunomodulatory, antidiabetic, antihypertensive, and antioxidant properties^1–5^. Nutritional and functional value of milk, particularly Mediterranean buffalo milk (*Bubalus bubalis*), can also be ascribed to the presence of a constitutive betaine, δ-valerobetaine (δVB) (*N,N,N*-trimethyl-5-aminovaleric acid), along with short-chain acylcarnitines^6,7^. In ruminant milk and meat, δVB originates from a specific transformation of plant N^ε^-trimethyllysine (TML) by the rumen microbiota^6,8^. Although rumen microorganisms involved in the process are not yet known, it is likely that TML undergoes to oxidative deamination (Stickland reaction) where it is oxidatively deaminated and transformed into the corresponding carboxylic acid with one carbon atom less, corresponding to δVB^8^. The buffalo milk content of δVB, l-carnitine and short-chain acylcarnitine is positively influenced by production systems^9^, as observed in buffaloes maintained in a larger allocation (15 square meter/head compared to 10 square meter/head). Indeed, a lower stress associated with the ruminating time typically higher in buffalo compared to cattle, confers a higher antioxidant and anti-inflammatory activity to the milk and a higher content of δVB, l-carnitine, acetyl-l-carnitine, propionyl-l-carnitine, and glycine betaine.

In human and mouse heart tissues, δVB regulates energy metabolism by inhibiting fatty acids β-oxidation with an effect similar to that of meldonium, a drug known to improve cardiac mitochondrial function after ischemia^10,11^. Moreover, results from the HealthGrain dietary intervention study showed that increased levels of δVB and other betainized compounds in fasting plasma were associated with improved insulin resistance and insulin secretion during diets rich in whole grains^10^. *In vitro* evaluation of the bioactivity of pure δVB and milk δVB showed effectiveness in the protection of endothelial cells against hyperglycemia-induced cell damage by counteracting intracellular reactive oxygen species (ROS) accumulation, cytokine release and downregulation of SIRT1 and SIRT6^12,13^.

The nuclear protein SIRT6 exerts diverse cancer-associated functions by controlling energy metabolism and stress resistance^14–16^. SIRT6 displays dual functions in tumorigenesis acting as tumor suppressor or promoter^15,17^. In fact, downregulation of SIRT6 expression relates to poor prognosis in human colorectal, breast, ovarian, lung, and pancreatic tumors, whereas in other tumors poor outcomes are associated to its overexpression^15,17^. Downregulated SIRT6 and upregulated nicotinamide mononucleotide adenylyltransferase 2 are associated with the presence, depth invasion, stage, and differentiation grade of colorectal cancer (CRC)^18^. SIRT6 phosphorylation by PKCζ at threonine 294 residue mediates fatty acid β-oxidation^19^ in human colon cancer cell lines, HCT116 and LoVo cells. Moreover, overexpression of SIRT6 in the SW480 CRC cell line induces G0/G1 phase arrest and represses the expression of the oncogenic cell division cycle 25 A phosphatase, supporting the suppressive role of SIRT6 in CRC^20^. On the other hand, downregulation of SIRT6 expression in colon cancer tissues negatively correlated with the overall survival of colon cancer patients^21^. The inhibitory effect of SIRT6 on colon cancer progression involves upregulation of PTEN, a major tumor suppressor of colon carcinogenesis, and potentiation of both SIRT6- and p53-mediated suppression of the oncogene c-myc^21,22^.

CRC, one of the most common malignant neoplasms in developed countries, is the second most diagnosed type of cancer in women and the third most common cancer in men with a mortality rate still unacceptably high^23^. Epidemiological and prospective studies have underlined the link between CRC etiology and modifiable lifestyle factors, such as diet. An inverse association between consumption of total milk with CRC risk has been observed^24,25^, as well as a negative association between the consumption of total dairy and the risk of CRC^26,27^. The risk of CRC has been reported to decrease by approximately 17% with increasing intake of dairy up to 400 g/d^28^.

In recent years, the use of natural drugs for CRC prevention has attained remarkable attention shifting the focus on toward effective preventive strategies with plant derived phytochemicals and functional metabolites of food origin which can effectively contribute to lower the cancer risk^29–31^. The chemopreventive role of dietary components in CRC, such as resveratrol, curcumin, quercetin, α-mangostin, ω-3-polyunsaturated fatty acids, vitamin D and dietary fiber has been reported to occur through the modulation of epigenetic regulators affecting cell proliferation/apoptosis, activating tumor suppressor genes (p53 and PTEN), and inducing ROS-mediated cytotoxicity^32^. Overall, although dietary phenolics are the most promising as possible future adjuvant in CRC management, the gap between preclinical and clinical research still exists since the amounts needed to exert some effects largely exceed common dietary doses. In this contest, exploring the anticancer properties of compounds occurring in highly consumed foods, such as milk, could represent a promising avenue in the search of naturally occurring biomolecules. The present study was designed to investigate the anti-neoplastic activity of a milk extract enriched with δVB in human colorectal adenocarcinoma. To this end, this study was conducted on HT-29 and LoVo cell lines showing APC/RAS (LoVo) and p53 (HT-29) mutations, known to be critical in the development of CRC via increasing adenomatous dysplasia.

## Results

### Effects of δVB and milk on cell viability

**Figure 1:**
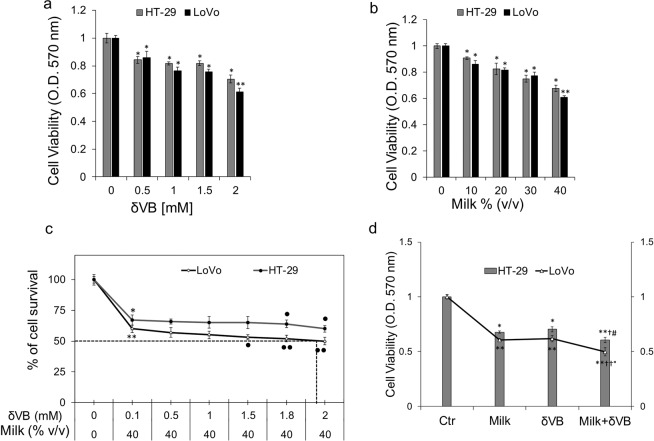
Inhibition of colorectal adenocarcinoma cell viability by milk-δVB. HT-29 and LoVo cells were treated with (**a**) increasing concentrations of δVB (up to 2 mM) or (**b**) increasing volumes of milk (up to 40% v/v) for 72 h. (**c**) Cell viability was determined after treatment with milk (40% v/v) enriched with serial concentrations of δVB (0.1, 0.5, 1, 1.5, 1.8 and 2 mM). After 72 h incubation, the IC_50_ was reached at the concentration of δVB 1.972 mM. IC_50_ values were calculated using GraphPad. (**d**) Colon cells were incubated for 72 h with 40% v/v milk, δVB (2 mM), or milk supplemented with δVB (milk + δVB). Control cells were grown in medium containing the same volume (% v/v) of HBSS-10 mM Hepes. Cell growth inhibition was assessed using MTT assay. Values represent the mean ± SD of three independent experiments. *P < 0.05 *vs* Ctr, **P < 0.01 *vs* Ctr, ^†^*P* < 0.05 *vs* milk, ^††^*P* < 0.01 *vs* milk, ^#^*P* < 0.05 *v*s δVB, *°P* < 0.01 *v*s δVB ^●^*P* <  0.05 *v*s 0.1 mM δVB, and ^●●^*P*  <  0.01 *v*s 0.1 mM δVB.

The cytotoxic effect of δVB was evaluated in CCD 841 CoN, HT-29 and LoVo cells for 24, 48 and 72 h. Results showed a time- and dose-dependent capability of δVB to inhibit selectively the viability of colon cancer cells, with highest potency observed in LoVo cells after 72 h of incubation with 2 mM δVB (*P* < 0.01) (Fig. 1a and Supplementary Fig. S1). In contrast, non-malignant CCD 841 CoN cells were only minimally affected by δVB and milk treatment after 72 h (15.5% and 14.8% inhibition of cell proliferation, respectively) (Supplementary Fig. S1). Despite the δVB content in buffalo milk is lower than 2 mM (about 106 μmol/L), 40% (v/v) of milk extract induced cytotoxicity in HT-29 and LoVo cell lines. However, LoVo cells were more responsive to milk extract, reaching the highest reduction in cell viability after 72 h of incubation (*P* < 0.01) (Fig. 1b and Supplementary Fig. S1). It emerged that LoVo cells responded to the combined treatment with δVB (2 mM) and milk (40% v/v) reaching the IC_50_ with 50.2% of cell viability inhibition (Fig. 1c). In the combined treatment (milk + δVB), the effects displayed by milk and δVB alone on cell viability were potentiated (*P* < 0.05 *vs* milk in HT-29 and *P* < 0.01 *vs* milk in LoVo) (Fig. 1d). Based on these results, LoVo cells were chosen for further experiments.

### Cell cycle modulation

**Figure 2:**
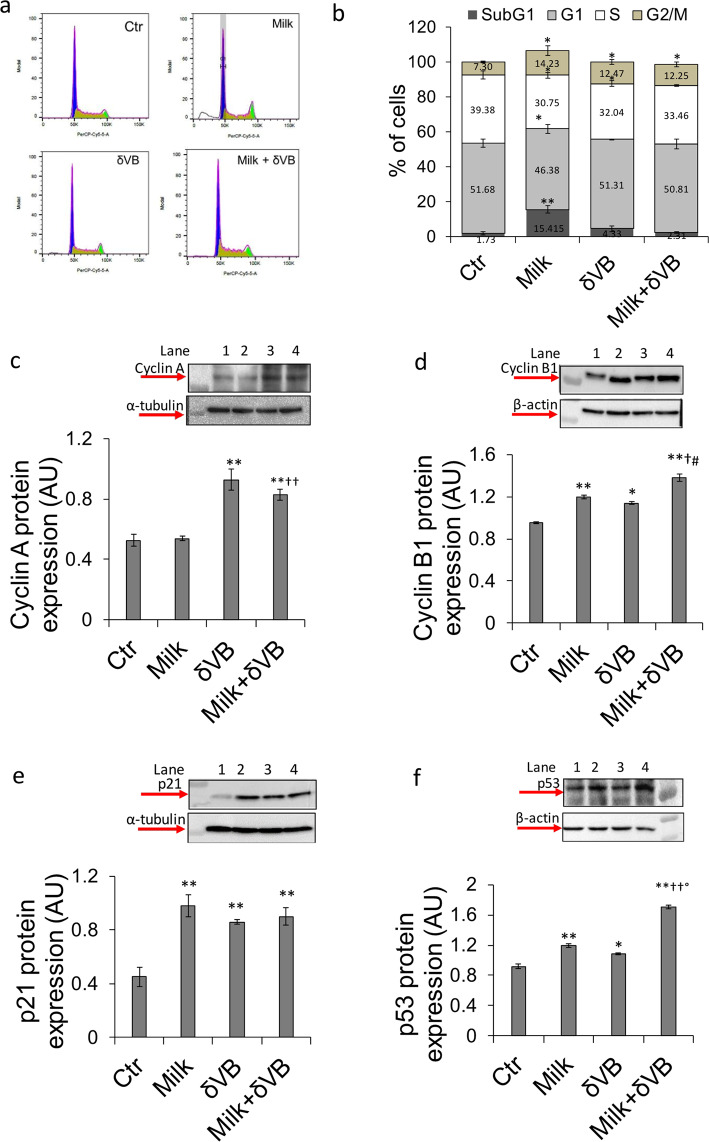
LoVo cell cycle alteration induced by milk-δVB. (**a,b**) Representative cell cycle analysis and average of LoVo cell cycle distribution. LoVo cells were treated with milk (40% v/v), δVB (2 mM), milk enriched with δVB (milk + δVB), or HBSS-10 mM Hepes (40% v/v) (Ctr) for 72 h. Cell cycle distribution was assessed by flow cytometry collecting PI fluorescence as FL3-A (linear scale) and analysis by ModFIT software (Verity Software House, USA, Becton Dickinson) (www.vsh.com/products/mflt/). For each sample at least 10.000 events were analyzed. (**c**–**f**) Representative images of Western blotting analysis of cyclin A, cyclin B1, p21 and p53 in LoVo cells after 72 h of treatment. Lane 1 = Ctr, lane 2 = milk, lane 3 = δVB, lane 4 = milk + δVB. Protein determination was performed with Image J software 1.52n version and quantified using α-tubulin or β-actin. Values are expressed as arbitrary units (AU). *P < 0.05 *v*s Ctr; **P < 0.01 *vs* Ctr; ^†^*P* < 0.05 *vs* milk, ^††^*P* < 0.01 *vs* milk, ^#^*P* < 0.05 *v*s δVB, *°P* < 0.01 *v*s δVB. The full-length blots are showed in the supplementary information (Fig. S2).

Cell cycle distribution analysis of LoVo cells treated with milk (40% v/v) showed an arrest in G2/M phase (14.23% *vs* 7.3% in control cells, *P* < 0.05). The same effect was induced by treatment with δVB (12.47% *vs* 7.3% in control cells, *P* < 0.05) (Fig. 2a,b**)**. A consistent accumulation of cells in SubG1 phase (15.415 ± 2.1 *vs* 1.73 ± 1.1%, *P* < 0.01) was observed only in the presence of milk treatment. The modulation of cell cycle phases by milk and δVB involves, at least in part, the upregulation of cyclin B1, p21, and p53 protein expressions, whereas cyclin A resulted to be upregulated only by δVB (Fig. 2c–f). Enrichment of milk with δVB potentiated the upregulation of cyclin A (*P* < 0.01 *vs* milk), cyclin B1 (*P* < 0.05 *vs* milk, *P* < 0.05 *vs* δVB), and p53 (*P* < 0.01 *vs* milk, *P* < 0.01 *vs* δVB) protein expressions compared to milk or δVB given alone.

### Autophagy induction

**Figure 3:**
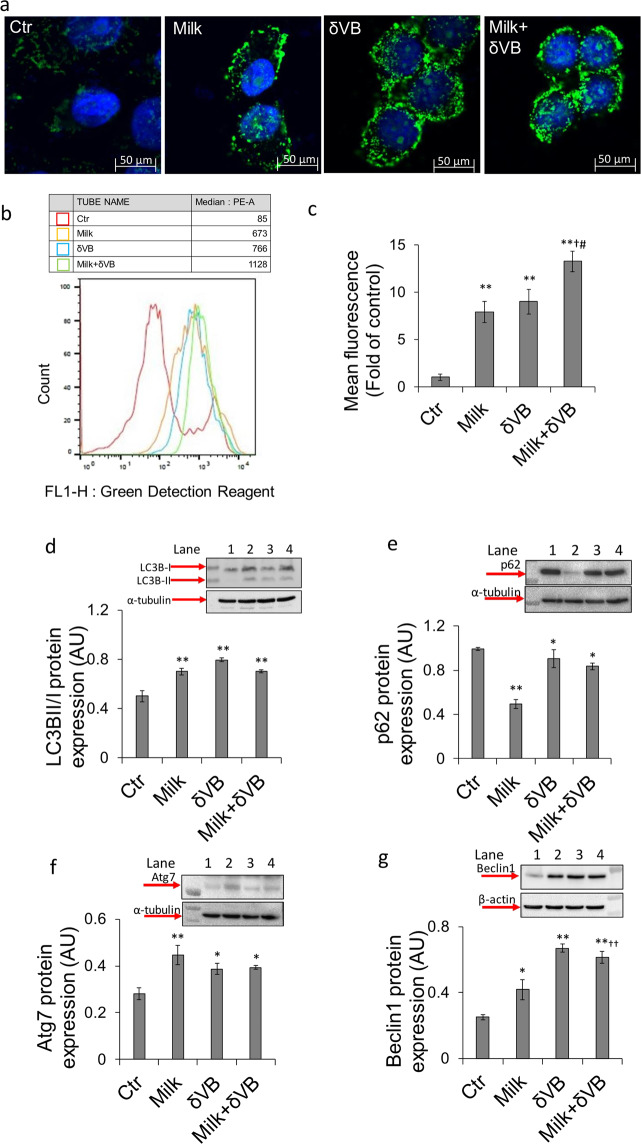
Autophagy induced by milk-δVB. (**a**) Representative images of green detection reagent imaged by confocal laser microscopy after 72 h in LoVo cells incubated with 40% v/v buffalo milk (milk), δVB (2 mM) and milk supplemented with δVB (milk + δVB). Scale bar, 50 μm. (**b**) As for flow cytometry analysis, at least 10.000 events were acquired in log mode. For the quantitative evaluation of green detection reagent, FlowJo V10 software was used to calculate median fluorescence intensities. (**c**) Bar graph of the fluorescence intensity values of green detection reagent-labeled vesicles and expressed as fold change of the control (Ctr). Analysis was carried out by determinations in triplicate of n = 3 experiments **P < 0.01 *vs* Ctr, ^†^*P* < 0.05 *vs* milk, ^#^*P* < 0.05 *vs* δVB. (**d**–**g**) Protein expression levels of LC3BII/ LC3BI, p62, Atg7 and Beclin 1, measured by Western blot in control LoVo cells (Ctr) or cells treated for 72 h with milk (40% v/v), δVB (2 mM) and milk+δVB. Lane 1 = Ctr, lane 2 = milk, lane 3 = δVB, lane 4 = milk + δVB. Protein content was calculated with Image J software 1.52n version and expressed as arbitrary units (AU). *P < 0.05 *vs* Ctr; **P < 0.01 *vs* Ctr; ^††^*P* < 0.01 *vs* milk. The full-length blots are showed in the supplementary information (Fig. S3).

Since milk and δVB were able to induce perturbations in cell cycle by affecting the expression of cyclin A, cyclin B1, p21and p53, we next investigated cell death mechanisms by evaluating autophagy occurrence. Results indicated that milk and δVB induced a 6-fold increase of autophagosome formation (Fig. 3a–c). This effect was enhanced by milk+δVB (1128 ± 13.79 mean fluorescence intensity, *P* < 0.05 *vs* milk, *P* < 0.05 *vs* δVB), thereby supporting the contribution of δVB to this cellular process. Positive controls were performed by treating LoVo cells with rapamycin (1 µM) for 16 h (Supplementary Fig. S3). Immunoblotting of autophagy marker proteins showed the accumulation of LC3BII, the lipidized form of LC3 correlating with the autophagosome formation (1.5-fold, *P* < 0.01 *vs* Ctr) (Fig. 3d,e). This effect was paralleled by the modulation of autophagic effectors, p62, Atg7 and Beclin1. In fact, treatment with milk inhibited p62 protein expression by about 50% (*P* < 0.01 *vs* Ctr) and increased the expression of Atg7 (*P* < 0.01 *vs* Ctr) and Beclin1 (*P* < 0.05 *vs* Ctr). LoVo cells responded to treatment with δVB and milk+δVB with a lower modulation of p62 and Atg7 protein expression (*P* < 0.05 *vs* Ctr). On the contrary, Beclin1 upregulation was triggered more markedly by δVB (*P* < 0.01 *vs* Ctr) and milk+δVB (*P* < 0.01 *vs* Ctr, *P* < 0.01 *vs* milk) (Fig. 3f,g).

### Modulation of SIRT6 protein

**Figure 4:**
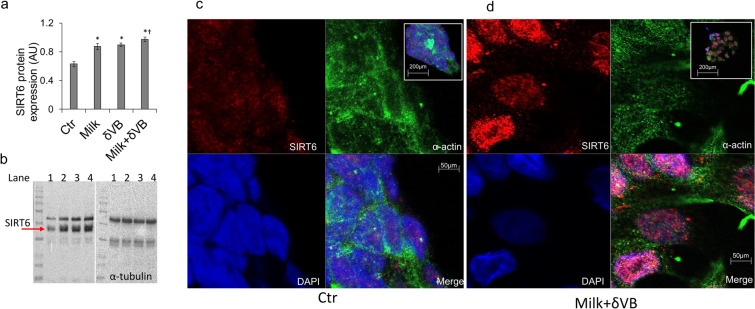
Upregulation of SIRT6 protein expression. (**a,b**) SIRT6 protein expression levels expressed as arbitrary units (AU) with *P < 0.05 *vs* Ctr, ^†^*P* < 0.05 *vs* milk. Lane 1 = Ctr, lane 2 = milk, lane 3 = δVB, lane 4 = milk + δVB. (**c,d**) Representative confocal images of SIRT6 expression (red) and α-actin (green) in control cells (Ctr) and cells exposed to milk+ δVB for 72 h. Nuclei were counterstained with DAPI (blue). Scale Bar, insert = 200 μm; Enlarged = 50 μm.

Evaluation of the possible involvement of SIRT6 indicated a positive regulation of SIRT6 protein expression levels following milk and δVB treatments (*P* < 0.05 *vs* Ctr), with an enhanced effect elicited by treatment with milk+δVB (*P* < 0.05 *vs* Ctr*, P* < 0.05 *vs* milk) (Fig. 4a,b). Cellular localization and upregulation of SIRT6 protein expression in LoVo cells treated with milk+δVB was also evidenced by confocal laser scanning microscopy analyses (Fig. 4c,d).

### Apoptosis triggered by milk and δVB

**Figure 5:**
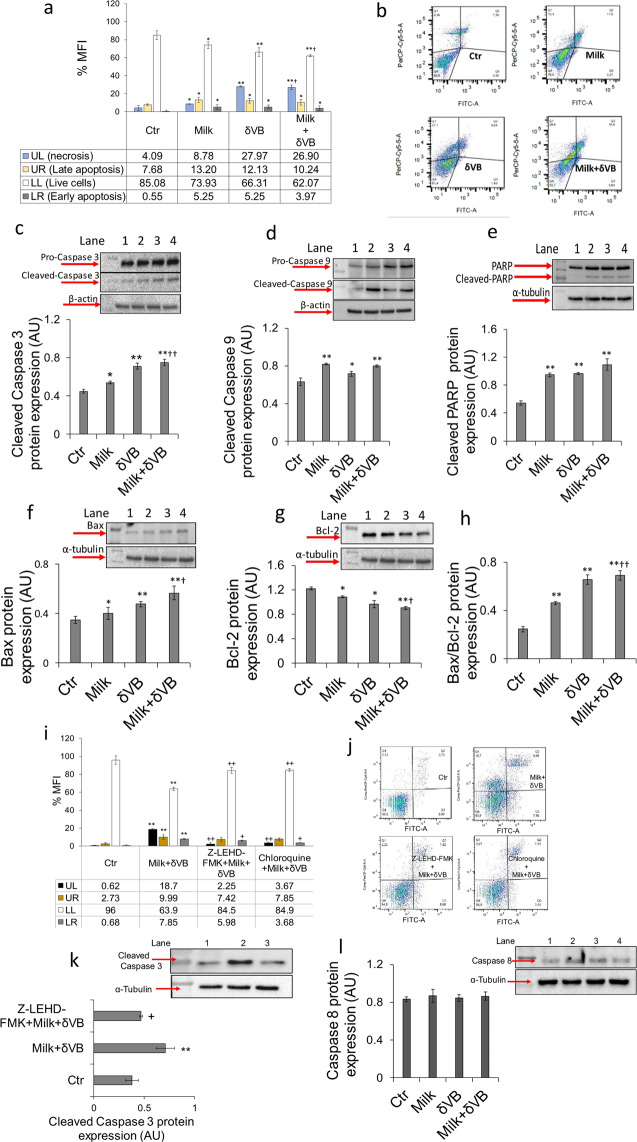
Apoptotic mechanism. (**a,b**) Percentage of apoptosis and representative dot plots of annexin V-FITC and PI-stained cells analyzed by flow cytometry. Data are expressed as mean ± SD of n = 4 experiments. At least 10.000 events were acquired. (**c**–**h**) Protein expression levels of caspase 3, caspase 9, PARP, Bax and Bcl-2 from LoVo cells treated for 72 h with milk (40% v/v), δVB (2 mM), milk+δVB, or HBSS-10 mM Hepes (40% v/v) (Ctr). Lane 1 = Ctr, lane 2 = milk, lane 3 = δVB, lane 4 = milk + δVB. Analysis of densitometric intensity was calculated with Image J 1.52n version software. Arbitrary units of protein expression (AU) were quantified using α-tubulin or β-actin. Antibodies against Bax, Bcl-2 and SIRT6 (reported in Fig. 4) were blotted on the same filter and quantified by using the same loading control (α-tubulin). (**i,j**) Flow cytometric analysis and representative dot plots of annexin V-FITC and PI double staining LoVo cells treated with caspase 9 inhibitor Z-LEHD-FMK (40 μM) or chloroquine (50 μM). Data are expressed as mean ± SD of n = 3 experiments. At least 10.000 events were acquired. (**k**) Cleaved caspase 3 protein expression level from LoVo cells treated for 72 h with milk+δVB, Z-LEHD-FMK + milk+δVB or HBSS-10 mM Hepes (40% v/v) (Ctr). Lane 1 = Ctr, lane 2 = milk + δVB, lane 3 = Z-LEHD-FMK + milk+δVB. (**l**) Protein expression levels of caspase 8 in LoVo cells after 72 h of treatment with milk (40% v/v), δVB (2 mM), milk+δVB, or HBSS-10 mM Hepes (40% v/v) (Ctr). Lane 1=Ctr, lane 2 = milk, lane 3 = δVB, lane 4 = milk + δVB. *P < 0.05 *vs* Ctr, **P < 0.01 vs Ctr, ^†^*P* < 0.05 *vs* milk, ^††^*P* < 0.01 *vs* milk, ^+^*P* < 0.05 *vs* milk+δVB, ^++^*P* < 0.01 *vs* milk+δVB. The full-length blots are included in the supplementary information (Fig. S4).

Flow cytometry analysis indicated that milk determined a decrease of cell viability (*P* < 0.05 *vs* Ctr) with an increase of late (*P* < 0.05 *vs* Ctr) ad early apoptosis (*P* < 0.05 *vs* Ctr) (Fig. 5a,b). Likewise, δVB determined a more marked reduction of viable cells (*P* < 0.01 *vs* Ctr) and increased cells in late and early apoptosis (*P* < 0.05 *vs* Ctr), as well. Noteworthy, δVB increased necrosis (*P* < 0.01 *vs* Ctr) and in the combined treatment with milk (milk + δVB) improved the effect of milk on live cell reduction (*P* < 0.05 *vs* milk, *P* < 0.01 *vs* Ctr) and necrosis (*P* < 0.05 *vs* milk) (Fig. 5a,b).

To further investigate the molecular mechanism(s) by which milk and δVB induced LoVo cell apoptosis, we next evaluated the expression of caspase 3 and caspase 9. We found that pro-caspase 3 and pro-caspase 9 proteins were significantly upregulated in cells treated with milk or δVB compared to cells treated with vehicles (Fig. 5c,d). However, only the upregulation of caspase 3 was further enhanced by milk+δVB (*P* < 0.01 *vs* milk). In addition, the cleavage of the mitochondrial apoptotic protein, poly (ADP-ribose) polymerase (PARP), increased following milk or δVB treatment (*P* < 0.01) (Fig. 5e). Molecular mechanism underlying apoptosis involved the modulation of Bax and Bcl-2 (Fig. 5f,g), as indicated by the increased Bax/Bcl-2 ratio in cells exposed to milk+δVB (*P* < 0.01 *vs* milk) (Fig. 5h). In this regard, co-treatment with caspase 9 inhibitor (Z-LEHD-FMK) and milk+δVB for 72 h reduced the apoptotic cell death (Fig. 5i,j**)** and inhibited the expression of cleaved caspase 3 protein (*P* < 0.05 *vs* milk+δVB) (Fig. 5k**)**, thus supporting the evidence that apoptosis occurs *via* intrinsic pathway. In addition, unchanged expression of caspase 8, the initiator caspase of extrinsic apoptosis, was observed during treatments (Fig. 5l). Finally, cell death induced by milk+δVB was blocked by the lysosomal inhibitor, chloroquine, suggesting that LoVo cell apoptosis occurs *via* autophagy (*P* < 0.01 *vs* milk in late apoptosis and *P* < 0.05 *vs* milk in early apoptosis) (Fig. 5i,j).

### δVB induced ROS accumulation

**Figure 6:**
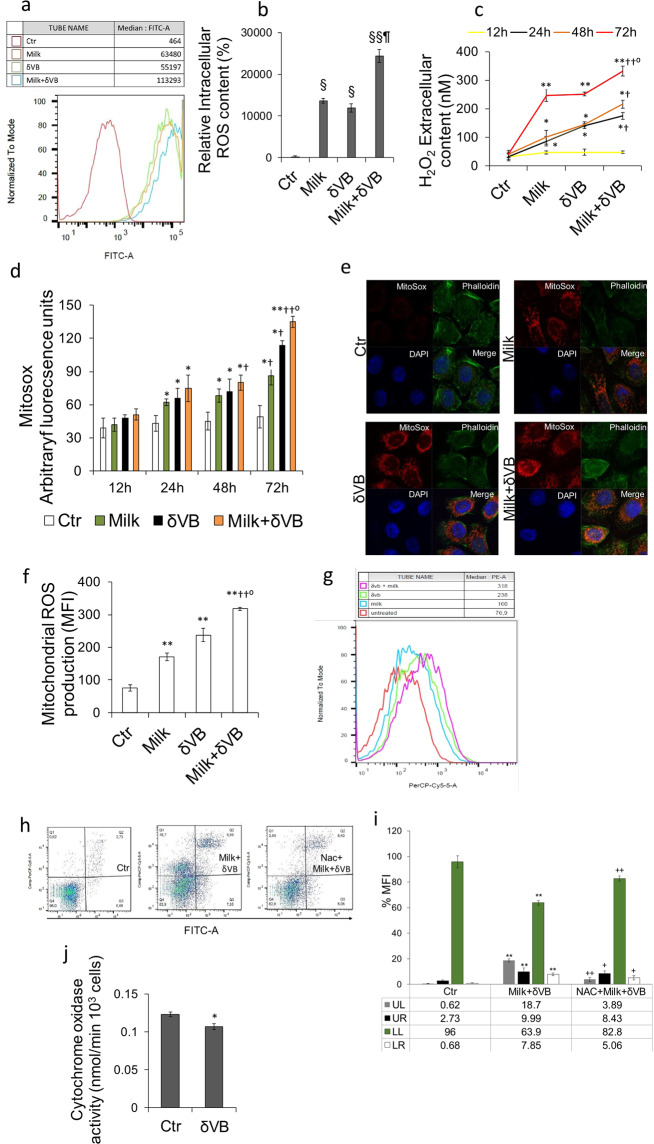
Evaluation of redox homeostasis. (**a,b**) Flow cytometry analysis of intracellular ROS levels after incubation of LoVo with 10 µM DCFH-DA was performed as described under Method section. (**c**) Time-line (12, 24, 48, and 72 h) of extracellular H_2_O_2_ production was determined with Amplex Red H_2_O_2_/peroxidase assay. Data are shown as mean ± SD of three independent experiments. (**d)** Time-dependent mitochondrial superoxide levels assessed in LoVo cells by MitoSOX-confocal microscopy analysis after 12, 24, 48, and 72 h of treatment with milk (40% v/v), δVB (2 mM), milk + δVB, or HBSS-10 mM Hepes (40% v/v) (Ctr). (**e)** Representative confocal images of MitoSOX (red) and phalloidin (green) in control cells (Ctr), milk (40% v/v), δVB (2 mM) or milk + δVB for 72 h. Nuclei were counterstained with DAPI. Scale bar, 50μm. (**f,g)** MitoSOX-based flow cytometry in cells treated with vehicle (Ctr), milk (40% v/v), δVB (2 mM) or milk + δVB, for 72 h in serum-free medium. Results are expressed as mean fluorescence intensity (MFI). (**h,i)** Percentage of apoptosis and representative dot plots of annexin V-FITC and PI-stained cells analyzed by flow cytometry in LoVo cells treated for 72 h with HBSS-10 mM Hepes (40% v/v) (Ctr) milk+ δVB, or NAC + milk+ δVB. Data are expressed as mean ± SD of n = 3 experiments. At least 10.000 events were acquired. **(j)** The effect of 72 h treatment with δVB (2 mM) on mitochondrial function was determined by assaying cytochrome oxidase activity in whole LoVo lysates. ^*^P < 0.05 *vs* Ctr, **P < 0.01 vs Ctr, ^††^*P* < 0.01 *vs* milk, *°P* < 0.01 *v*s δVB, ^§^*P* < 0.001 *vs* Ctr; ^§*§*^*P* < 0.0001 *vs* Ctr, ^¶^*P* < 0.001 *vs* milk, ^+^*P* < 0.05 *v*s milk+δVB, ^++^*P* < 0.01 *vs* milk+δVB.

Evaluation of cellular redox status was performed by three independent approaches to determine intracellular ROS, extracellular H_2_O_2_, and mitochondrial ROS. Results showed an increased intracellular ROS generation during 72 h of treatment with milk and δVB up to 137- and 120-fold, respectively (*P* < 0.001 *vs* Ctr) (Fig. 6a,b), with a synergistic effect in cells exposed to milk+δVB (*P* < 0.0001 *vs* Ctr, *P* < 0.001 *vs* milk). The fluorescent signal observed in cells treated with menadione (100 µM) confirmed that the signal was specifically produced by the increase of ROS (Supplementary Fig. S5a). Intracellular ROS elevation was not observed in normal CCD 841 CoN cells, suggesting that ROS induction is specific for cancer cells (Supplementary Fig. S5d,e).

Extracellular ROS production measurement with Amplex Red assay indicated that exposure to milk or δVB determined an overtime increase of extracellular H_2_O_2_ starting from 24 h and continuing up to 72 h (*P* < 0.01 *vs* Ctr). This effect was even more pronounced in cells exposed to milk+δVB (*P* < 0.001 *vs* Ctr, *P* < 0.01 *vs* milk and δVB alone) (Fig. 6c). We next evaluated mitochondrial ROS levels and localization using the chemical probe MitoSox (Fig. 6d–g and Supplementary Fig. S5b,c). Results showed that mitochondrial ROS production was activated by milk (2.5- fold change) and δVB (3-fold change) (*P* < 0.01 *vs* Ctr). The increase of MitoSox fluorescence was more significant in milk+δVB treated cells (3.2-fold) (*P* < 0.001 *vs* Ctr, *P* < 0.01 *vs* milk). Suppression of ROS generation by pre-treatment with NAC reduced the pro-cell death effects, confirming that the production of ROS induced by milk-δVB triggers the apoptotic cell death **(**Fig. 6h,i**)**. In particular, an increase of viable cells occurred following treatment with NAC + milk+δVB (82.8 ± 2.09% *vs* 63.9 ± 1.85% in milk+δVB, *P* < 0.01), along with a decrease of late (8.43 ± 1.09% *vs* 9.99 ± 1.14% in milk+δVB, *P* < 0.05) and early apoptosis (5.06 ± 0.73% *vs* 7.85 ± 0.91% in milk+δVB, *P* < 0.05). Of note, NAC pre-treatment strongly counteracted the necrotic process (3.89 ± 1.67% *vs* 18.7 ± 3.09 in milk+δVB, *P* < 0.01) **(**Fig. 6h,i**)**.

**Figure 7:**
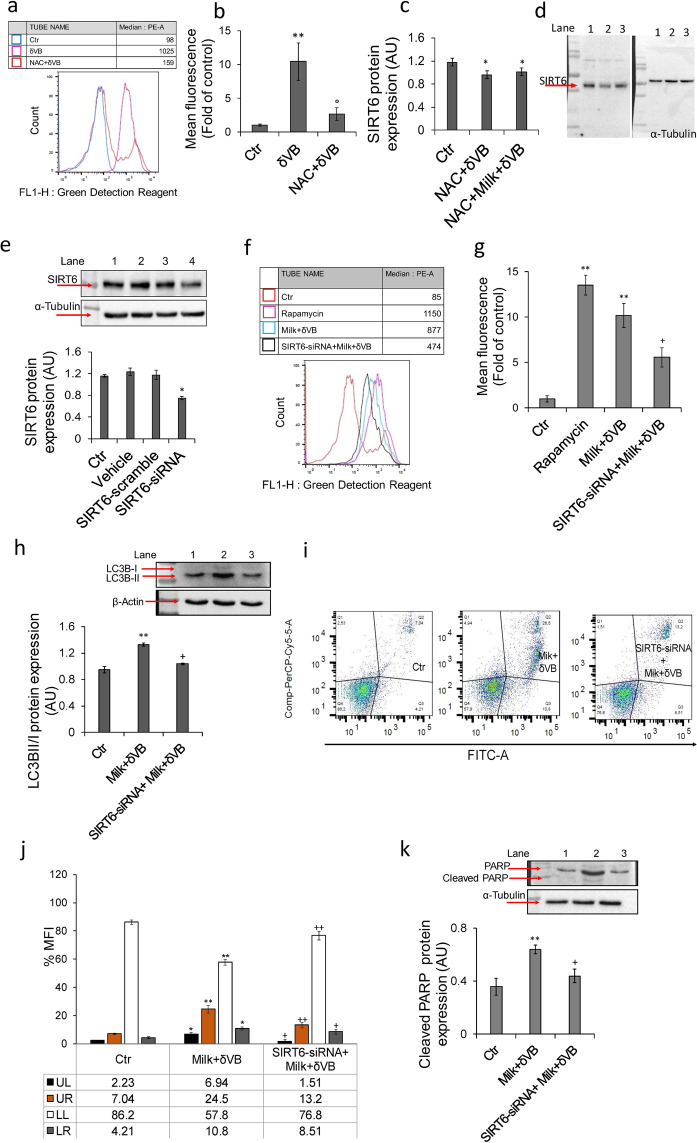
Suppression of ROS production. (**a,b**) Flow cytometry analysis of green detection reagent staining following incubation with δVB (2 mM) and δVB + NAC. **P < 0.01 *vs* Ctr, *°P* < 0.01 *v*s δVB. (**c, d**) Arbitrary units (AU) of SIRT6 protein expression in cells treated with δVB and milk+δVB in the presence of NAC. Lane 1 = Ctr, lane 2 = NAC + δVB, lane 3 = NAC + milk + δVB, *P < 0.05 *vs* Ctr. (**e**) SIRT6 protein expression in c**e**lls treated with RNAifectin transfection reagent (Vehicle), scramble siRNA (50 nM) (Scramble), SIRT6-siRNA (50 nM) or medium only (Ctr). Lane 1 = Ctr, lane 2 = Vehicle, lane 3 = Scramble, lane 4 = SIRT6-siRNA. (**f,g**) Flow cytometric autophagic activity performed by green detection reagent in LoVo cells treated for 72 h with milk+δVB, SIRT6-siRNA+milk+ δVB, or HBSS-10 mM Hepes (40% v/v) (Ctr). Rapamycin (1 μM) was used as positive control. FlowJo V10 software was used to calculate median fluorescence intensities. (**h)** Protein expression levels of LC3BI/ LC3BII from LoVo cells treated for 72 h with milk + δVB, SIRT6-siRNA + milk + δVB, or HBSS-10 mM Hepes (40% v/v) (Ctr). Lane 1 = Ctr, lane 2 = milk + δVB, lane 3 = SIRT6-siRNA + milk + δVB. (**i,j**) Percentage of apoptosis and representative dot plots of annexin V-FITC and PI-stained cells analyzed by flow cytometry in LoVo cells after 72 h of treatment with milk+δVB, SIRT6-siRNA+milk+δVB, or HBSS-10 mM Hepes (40% v/v) (Ctr). Data are expressed as mean ± SD of n = 4 experiments. At least 10.000 events were acquired. (**k**) Protein expression levels of PARP from LoVo cells treated for 72 h with milk + δVB, SIRT6-siRNA + milk + δVB, or HBSS-10 mM Hepes (40% v/v) (Ctr). Lane 1 = Ctr, lane 2 = milk + δVB, Lane 3 = SIRT6 siRNA + milk + δVB. *P < 0.05 *vs* Ctr, **P < 0.01 *vs* Ctr, ^+^*P* < 0.05 *vs* milk + δVB, ^++^*P* < 0.01 *vs* milk + δVB.

To investigate the specific role of δVB in the mechanism of cellular ROS accumulation, further experiments were designed to evaluate the mitochondrial function in cells exposed to δVB for 72 h. Results indicated that the cytochrome oxidase activity was inhibited by δVB (*P* < 0.05 *vs* Ctr) (Fig. 6j). Intriguingly, blocking the intracellular ROS with the antioxidant NAC reduced the autophagy induced by δVB (*P* < 0.01 *vs* δVB), suggesting that the apoptotic cell death is likely to occur through changes in mitochondrial integrity initiated by excessive ROS generation (Fig. 7a,b). Moreover, results showed that ROS generation by milk-δVB correlates with upregulation of SIRT6 protein expression which was reduced by co-treatment with NAC (Fig. 7c,d).

### SIRT6 mediated LoVo cell death

The specific role of SIRT6 in LoVo cell death was next evaluated by transient silencing of *SIRT6* gene carried out with small interfering RNA (SIRT6-siRNA) (Fig. 7e**)**. Results indicated that SIRT6-siRNA transfection reverted the effects of milk+δVB on autophagy (1.8-fold decrease *vs* milk+δVB, *P* < 0.05) (Fig. 7f,g**)** and LC3BII protein upregulation (*P* < 0.05 *vs* milk+δVB) (Fig. 7h). In addition, SIRT6-siRNA determined an increase of viable cell (*P* < 0.01 *vs* milk+δVB), a decrease of late (*P* < 0.01 *vs* milk+δVB) and early apoptosis (*P* < 0.05 *vs* milk+δVB), and a 4.6-fold decrease of necrosis (*P* < 0.05 *vs* milk+δVB) (Fig. 7i,j**)**. The inhibition of milk+δVB induced apoptosis by SIRT6-siRNA occurred via downregulation of total and cleaved PARP expression levels (*P* < 0.05 *vs* milk+δVB) (Fig. 7k). On the whole, these results strengthened the evidence about the role of SIRT6 in the apoptotic LoVo cell death driven by autophagy.

## Discussion

The present study provides the first evidence of the cytotoxic effect of δVB in HT-29 and LoVo colon cancer cells, with a highest potency displayed in LoVo cells. The apoptotic effects of milk extracts and milk extracts enriched with δVB highlighted a specific role of δVB in these cellular processes. The possible mechanism underlying the anti-proliferative effects is likely due to cell-cycle related cyclins and regulatory proteins, p21^Cip1^ and p53. Moreover, we also demonstrated that the reduction of cell proliferation involved molecular processes and signaling pathways controlling autophagy, due to an increased level of beclin and a necrotic rate in δVB-treated cells. Notably, the apoptotic cell death *via* caspase 3 and caspase 9 involves activation of SIRT6 and changes in mitochondrial integrity initiated by excessive ROS accumulation suggesting a redox-dependent mechanism.

Eating habits are believed to influence the risk of CRC. Current epidemiological and meta-analysis studies show convincing evidence of decreased risk of CRC following milk and dairy products consumption^24,26,33–36^. We previously reported that buffalo milk (*Bubalus bubalis*), a ruminant milk particularly rich in δVB, possesses high antioxidant and anti-inflammatory activities and displays beneficial effects against endothelial dysfunction induced by hyperglycaemia^7,9,13^. Here, a buffalo milk extract and δVB efficiently suppressed the viability of LoVo cells in a time- and concentration-dependent manner inducing a G2/M arrest, SubG1 accumulation, and apoptosis via p53-mediated cascades. Moreover, these effects were potentiated by milk-enrichment with δVB, pointing out the importance of multiple food components instead of single nutrients in potentiating the health-promoting properties of naturally occurring biomolecules.

Autophagy frequently occurs with cell death or precedes it. In the latter case, autophagic membranes or proteins control cancer cell growth by facilitating the activation of apoptosis or necrosis^37–39^. In the advanced stages of CRC, autophagy shows a decisive role in the activation of cellular signals required for the phagocytic engulfment of apoptotic cells^40^. The increased expression of LC3BII/I, a key regulator of autophagosome nucleation, Beclin 1, and Atg7 following treatment with milk-δVB suggested a raise of the autophagic flux, a process commonly activated by anti-cancer agents^41^. The evidence here provided that autophagy and apoptosis are simultaneously triggered by milk-δVB suggest a cell death finely regulated by a cross talk between autophagy and apoptosis^39^. This hypothesis is supported by the evidence that the lysosomal inhibitor, chloroquine, blocked the cell death indicating that autophagy is not a merely general stress response without any further consequences but drives apoptosis.

The activation of SIRT6 by milk-δVB is consistent with a recent report showing that pharmacological activation of SIRT6 triggers lethal autophagy in HCT116 human colon cells by enhancing LC3B conversion from LC3BI to the autophagosome-associating form, LC3BII^42^. SIRT6 has emerged as an important target for cancer prevention and treatment^17,43^. However, due to its dual role in cancer as both tumor suppressor and oncogene, the identification of conditions able to control SIRT6 regarding cancer prevention and treatment is challenging. In CRC, downregulation of SIRT6 predicts a poor prognosis and aggressiveness, suggesting that it might act as a tumor suppressor^22^. Moreover, SIRT6 downregulation in colon cancer tissues and different colon cancer cell lines negatively correlated with the overall survival of patients through the regulation of PTEN/AKT signaling pathway^21^. A more recent study performed on 50 patients with CRC showed a lower expression of SIRT6 compared to normal controls, whereas patients with higher SIRT6 level had a better prognosis^44^. However, the mechanism through which SIRT6 controls cancer progression is intriguing and, depending on the biologic context, both increased and reduced SIRT6 activity could be exploitable by cancer cells^14,17,45^. In this regard, SIRT6 mediates breast cancer cell cancer survival and oxidative stress resistance by regulating intracellular NAMPT activity and NAD(P)(H) levels, suggesting the use of SIRT6 inhibitors and agents inducing oxidative stress as a promising strategy for cancer treatment^45^.

Cancer cells operate under constant oxidative stress and are very sensitive to the disruption of their enhanced ability to scavenge free radicals. However, increased oxidative stress is likely to make cancer cells more vulnerable to damage by additional ROS insults induced by exogenous agents^46^. Milk and δVB, and even more δVB-enriched milk, determined a consistent intracellular, extracellular and mitochondrial ROS production, pointing out a role of δVB in the altered redox homeostasis. Our data indicate that ROS production induced by milk-δVB is not a secondary effect but it triggers LoVo cell death, as demonstrated by the reduced pro-cell death effects when ROS generation is suppressed by NAC antioxidant. Although the complete molecular  mechanism(s) linking the increased ROS production and SIRT6 activation was not here investigated, it is tempting to speculate that ROS burst induced by milk-δVB might affect the upstream events leading to autophagy commitment and that the increased activity of SIRT6 requires NAD + , mainly produced at the level of mitochondrial electron transport chain. Finally, it cannot be ruled out that colorectal metabolic stress induced by δVB might depend on a mechanism most likely common to L-carnitine^11^.

To date, δVB has been detected at micromolar concentrations in human and mouse heart tissues and has been shown to inhibit *in vitro* β-oxidation of fatty acids and high-glucose cytotoxicity at concentrations of 100 μM and 500 μM, respectively^10,11,13^. Although among ruminant milk buffalo milk shows the highest content of δVB (106 μmol/L)^7,9^, milk extract was enriched with pure δVB up to the final concentration of 2 mM. However, given the importance of additive and /or synergistic interactions of food components, here supported by the evidence of the highest potency of δVB-enriched milk, it cannot be ruled out that the interaction of δVB with other bioactive milk components could be more effective in inducing apoptosis or even a programmed form of necrosis or inflammatory cell death, such as necroptosis^47,48^. Our results give an *in vitro* proof of concept that altered redox homeostasis and activation of SIRT6 induced by milk-δVB are associated with the induction of intrinsic apoptosis, providing a rationale for the definition of innovative dietary interventions aimed to provide adequate amounts of biomolecules through foods with a high functional profile. In this regard, consumption of dairy products showed an association with decreased risk of cardiovascular disease and CRC and led to the hypothesis of their possible positive impact on health^33^. In particular, among specific types of dairy foods, the strongest evidence (probable) for decreased risk of cardiovascular disease and CRC has been associated with yoghurt and cheese^33^. The proposed mechanisms of CRC protection associated with dairy consumption involves the influence of vitamin D on calcium metabolism and the modulation of the production of gut microbiota genotoxin^33,49^. The consumption of milk and dairy products also affects the balance between tumorigenic metabolites, such as secondary bile acids, and the healthy metabolites of microbial production^50^. In this context, it is now evident that other mechanism(s) related to the betaines from dietary source can take part to the health-promoting properties of milk and dairy products, highlighting the importance of defining innovative breeding techniques aimed to improve the content of bioactive metabolites in milk.

Finally, our observations unveil the anti-neoplastic effects of milk-δVB suggesting a promising role as food-based preventive therapy for colon cancer. Further studies on a complete panel of CRC cell lines reflecting diverse stages of this malignancy are critical before approaching *in vivo* studies to deepen the potential of targeting SIRT6 or using redox-modulating strategies in the prevention of CRC.

## Methods

### Chemical and antibodies

δVB synthesis and purification was carried out as previously described^6^.

Kaighn’s Modification of Ham’s F-12 Medium (F-12K, 30-2004), Eagle’s minimum essential medium (EMEM, 30-2003), McCoy’s 5 A Medium (30-2007), fetal bovine serum (FBS, 30-2020) were obtained from American Type Culture Collection (ATCC, 10801 University Boulevard Manassas, Virginia). Penicillin-streptomycin and trypsin/EDTA were purchased from Gibco, Life Technologies (168 Third Avenue Waltham, MA USA). 3-[4,5-dimethylthiazol-2-yl]-2,5-diphenyltetrazolium bromide (MTT, M5655), 2′,7′-dichlorodihydrofluorescein diacetate (DCFH-DA, D6883), N-acetyl-L-cysteine (NAC) (A7250-25G), chloroquine (C6628) and menadione (M5625) were purchased from Sigma Aldrich (Via Monte Rosa, 93 20149 Milan IT). Amplex Red Peroxide/Peroxidase Assay Kit (A22188) and Mitosox Red Mitochondrial Superoxide Indicator (M36008) were bought from Thermo Fisher Scientific (Im Steingrund 4, 63303 DE). FITC Annexin V Apoptosis detection kit (556547) and immobilon Western Chemiluminescent HRP Substrate (WBKLS0100) were obtained from BD Pharmigen (2350 Qume Drive San Jose, CA) and Millipore (80 Ashby Rd, Bedford, MA 01730 USA), respectively. Antibody anti-p53 (orb323871), anti-cleaved-caspase 3 (orb106556) were bought from Biorbyt; Autophagy assay kit (ab139484), Rapamycin (ab139484); anti-SIRT6 (ab191385) and anti-β-actin (ab8227), anti-alpha tubulin (ab18251), Phalloidin-iFluor 488 Reagent (ab176753) were from Abcam (Cambridge CB2 0AX UK); anti-Bax (5023), anti-Bcl-2 (15071), anti-p21 (2947), anti-beclin-1 (4122), anti-cyclin A2 (4656), anti-caspase 3 (9662), anti-LC3B (2775), anti-p62 (SQSTM1) (5114), anti-cyclin B1 (4138), anti-caspase 9 (9508), anti-poly(ADP ribose) polymerase (PARP) (9532), anti-Atg7 (8558), anti- Caspase 8 (1C12) (9746); anti-α-tubulin (3873), anti-β-actin (3700) from Cell Signaling Technology (3 Trask Lane Danvers, MA, 01923 USA). Caspase 9 inhibitor, Z-LEHD-FMK (FMK008) was from R&D Systems. Secondary antibodies Alexa Fluor 488 and Alexa Fluor 633 were purchased from Life Technologies (168 Third Avenue Waltham, MA USA). RNAifectin transfection reagent (G073) SIRT6 siRNA oligos set (438110910101) and scramble siRNA were from Applied Biological Materials Inc (#1-3671 Viking Way Richmond, BC USA). All other solvents and reagents used were of analytical grade.

### Milk sample collection and preparation

Bulk milk was collected from Italian Mediterranean buffaloes (*Bubalus bubalis*) from Southern Italy and extracts were prepared as previously described^6,7^. To recover metabolites with low molecular weight, the central aqueous phase was filtered using Amicon Ultra 0.5 mL centrifugal filters (3 kDa molecular weight cutoff). Before being used, milk extracts were filtered through 0.22 μm Millipore filters. Determination of δVB content in milk was performed as previously described^6,7,9^.

### Cell culture and treatments

Human colon CCD 841 CoN cells (CRL-1790), HT-29 (HTB-38) and LoVo cells (CCL-229) were obtained from ATCC and grown in EMEM, McCoy’s 5 A and F-12K medium, respectively. Cells were maintained as a monolayer in a humidified incubator, 5% CO_2_, at 37 °C in specific culture medium supplemented with 100 U/mL penicillin, 100 mg/mL streptomycin and 10% FBS. The day before treatments cells were seeded into multi-well plates to allow cell attachment. Treatments were performed by culturing cells in complete medium with buffalo milk extracts (up to 40% v/v), δVB (up to 2 mM), or buffalo milk extracts enriched with δVB to a final concentration of 2 mM (milk + δVB) for a maximum time of 72 h. Control (Ctr) cells were treated with corresponding volumes (% v/v) of Hanks’ balanced salt solution (HBSS)-10 mM Hepes. When treated with NAC (5 mM) or menadione (100 µM) cells were pre-treated for 2 h and then for 72 h in the presence of milk and/or δVB. To perform a positive or negative control of autophagy process, LoVo cells were incubated with Rapamycin (1 µM) or Chloroquine (50 µM), respectively for 16 h before cytometric analysis. Caspase 9 inhibitor, Z-LEHD-FMK (40 µM) was added to LoVo cells before starting treatments.

### Cell viability assay

CCD 841 CoN, HT-29 and LoVo cells (2 × 10^3^ cells/well) were seeded in 96-well plates using the specific complete growth medium. After treatments, following manufacturer’s instruction, cell viability was determined by MTT assay. Absorbance at 570 nm and 695 nm was measured using a microplate reader model 680 Bio-Rad. All assays were performed with n = 6 replicates.

In order to determine the half maximal inhibitory concentration (IC_50_) of milk+δVB, LoVo cells were treated with milk (40% v/v) enriched by serial concentrations of δVB (0.1, 0.5, 1, 1.5, 1.8 and 2 mM) over a 72-hour period. Using linear regression, IC_50_ value of 1.972 mM for δVB in LoVo cells was calculated. For HT-29 cells milk+δVB enrichment up to 2 mM did not satisfy the IC_50_ achievement. Data analysis for IC_50_ determination was carried out using prism software program (Graph pad Software incorporated, version 6, www.graphpad.com/scientific-software/prism/).

### Cell cycle evaluation

LoVo cells (8 × 10^4^/well) were seeded in 6-well plates and after treatments were stained in a Propidium Iodide (PI) solution (50 μg/ml PI, 0.1% sodium citrate, 25 μg/ml RNase A, 0.1% triton in phosphate-buffered saline (PBS)) for 1 h in the dark. Flow cytometric analysis was performed using a BD Accuri C6 (Becton Dickinson, San Jose, CA). For the evaluation of intracellular DNA content, at least 10.000 events for each point were analyzed with the FlowJo V10 software (FlowJo LLC, USA) (www.flowjo.com/solutions/flowjo).

### Autophagy detection

LoVo cells (8 × 10^4^/well) were seeded in 6-well plates and after treatments green detection reagent, a cationic amphiphilic tracer (CAT), was added to each well (2% v/v in assay buffer), at 37 °C for 30 min in order to observe changes in autophagic flux. Cells were then detached and washed with PBS before analysis of fluorescence intensity by BD Accuri C6, by collecting the emissions as FL1-H (linear scale). Rapamycin (1 µM) was used as an autophagy inducer control. For confocal laser scanning microscopy analysis, LoVo cells (8 × 10^3^/well) were grown on coverslips and autophagic signal was analyzed by 63X oil immersion objective. Phalloidin-iFluor 488 reagent was used to identify actin filaments. Nuclei were counterstained with DAPI.

### Evaluation of apoptosis

Apoptotic (Annexin V-FITC-positive, PI-positive) from necrotic (Annexin V-FITC-negative, PI-positive) cells were distinguished by using FITC Annexin V Apoptosis detection kit. Briefly, treated cells were detached by trypsinization, washed with PBS and incubated in 200 μl binding buffer 1×, containing 2 μl Annexin V-FITC and 2 μl PI (20 μg/mL) for 30 min. Viable cells, early apoptotic cells, late apoptotic cells, and necrotic cells were detected by flow cytometry analysis (BD Accuri C6).

### Intracellular reactive oxygen species (ROS) measurement

Intracellular ROS levels were determined by 2′,7′-dichlorodihydrofluorescein diacetate (DCFH-DA) fluorescence staining following manufacturer’s instruction. The DCF fluorescence intensity was quantified using a BD Accuri C6 cytometer. Menadione (100 µM) was used as a positive control.

### Measurement of extracellular H_2_O_2_ using Amplex Red

Extracellular H_2_O_2_ released from LoVo cells after milk-δVB treatment was detected by Amplex Red Hydrogen Peroxide/Peroxidase Assay Kit. Briefly, after 72 h of treatment in serum-free medium, LoVo cells were washed with 1X PBS, trypsinized, and dispersed to produce a 20 µl cell suspension containing 2 × 10^4^ live cells in Krebs-Ringer phosphate glucose buffer (145 mM NaCl, 5.7 mM sodium phosphate, 4.86 mM KCl, 0.54 mM CaCl_2_, 1.22 mM MgSO_4_, 5.5 mM glucose, pH 7.35). The cells were mixed with 100 µl Amplex Red reagent containing 50 µM Amplex Red and 0.1 U HRP/ml in Krebs-Ringer phosphate glucose buffer and incubated at 37 °C for 60 min. The fluorescence of the oxidized 10-acetyl-3,7-dihydroxyphenoxazine was measured at an excitation wavelength of 530 nm and an emission wavelength of 590 nm, using a Tecan Infinite 2000 Multiplate reader. H_2_O_2_ was quantified with an H_2_O_2_ standard curve (0-2 µM concentration range).

### Mitochondrial ROS assessment

Mitochondrial ROS levels were measured using Mitosox Red Mitochondrial Superoxide Indicator following manufacturer’s protocol. Briefly, after 72 h of treatment in serum-free medium, cells were washed with pre-warmed 1X Hanks’ Balanced Salt Solution (HBSS) followed by staining with 5 μM Mitosox at 37 °C for 20 min. LoVo cells (8 × 10^3^/well) were seeded in 24-well plate containing microscope glass and imaged on confocal laser scanning microscopy analysis, as previously described.^13^For flow cytometric measurement, LoVo cells (0.5 × 10^6^) were harvested, suspended in 0.5 ml of complete medium containing MitoSOX (5 μM) and transferred to a shaking water bath (37 °C) protected from light for 20 min. The mean fluorescence intensity (MFI) was quantified using a BD Accuri C6 cytometer. Data were analyzed by FlowJo V10 software. Menadione (100 µM) was used to perform a positive control.

### SIRT6 (NM_016539) gene silencing

LoVo cells were transfected with small interfering RNA (siRNA) (50 nM) consistent of three SIRT6 siRNA oligos and with a scramble siRNA (50 nM) as control using RNAifectin transfection reagent. Transient transfection was performed following the manufacturer’s instructions. In detail, (2 × 10^5^ cells/well) were seeded in a six well tissue culture until reached 80% of confluence the day of transfection. After removing the growth medium, the transfection complexes (siRNA-RNAifectin) were added to serum-free and antibiotic-free medium. Finally, cells were incubated with transfection medium for 16 h before starting treatments.

### Protein extraction and Western blotting analysis

Cells were lysed in lysis buffer (1% NP-40, 0.5% sodium deoxycholate, 0.1% SDS in PBS) containing 10 μg/mL aprotinin, leupeptin and 1 mM phenylmethylsulfonyl fluoride (PMSF). 30-60 μg of sample proteins were separated by sodium dodecyl sulfate-polyacrylamide gel electrophoresis (SDS-PAGE) and then transferred to nitrocellulose membranes. Membranes were incubated for 1 h at room temperature (RT) with blocking buffer solution, TBS-T containing 20 mM Tris, pH 7.6, 100 mM NaCl, 0.1% Tween-20 and 5% non-fat dry milk under gentle shaker. Membranes were then incubated with specific primary antibodies at 4 °C overnight, followed by incubation with peroxidase-conjugated secondary antibodies for 1 h at RT. Primary antibodies used were anti-p53 (1:1000), anti-SIRT6 (1:1000), anti- PARP (1:1000), anti- Bax (1:1000), anti-Bcl-2 (1:1000), anti-p21 (1:500), anti-beclin-1 (1:1000), anti-cyclin A2 (1:1000), anti-caspase 3 (1:2000), anti-cleaved-caspase 3 (1:1000), anti-caspase 9 (1:1000), anti-caspase 8 (1:1000), anti-LC3B (1:1000), anti-p62 (1:3000), Atg7 (1:1000), and anti-cyclin B1 (1:1000). α-tubulin (1:5000) and β-actin (1:5000) were used for protein expression normalization. Host species (mouse or rabbit) of loading control were chosen based on primary antibody used. ECL detection was used to reveal immunocomplexes. Images were acquired by using Image Lab 5.2.1, Molecular Imager ChemiDoc XRS Imaging system (Bio-Rad Laboratories, Milan, Italy) and band densities were measured by ImageJ software 1.52n version (National Institutes of Health, Bethesda, USA) (https://imagej.nih.gov/ij/) and expressed as arbitrary units (AU).

### Cytochrome oxidase activity

Cytochrome oxidase activity was determined as previously described^51^. Briefly, 1 × 10^6^ treated cells were resuspended in 250 µl of a solution of 220 mM mannitol, 70 mM sucrose, 20 mM Tris-HCl, 1 mM EDTA, 5 mM EGTA (pH 7,4). Cell lysates were prepared by freezing and thawing three times. To unveil the enzyme cells were diluted 1:2 with the same buffer containing lubrol (1% w/vol) and then kept on ice for 30 min. Cytochrome oxidase activity was determined polarographically at 37 °C by using Oroboros 2k-Oxygraph system instrument^51^. 10^5^ cells were in incubated in 2 mL of reaction medium containing 30 µM cytochrome c, 4 µM rotenone, 0.5 mM dinitrophenol (DNP), 10 mM Na-malonate and 75 mM HEPES at pH 7.4^51^. After about 10 minutes the substrate [4 mM Na ascorbate with 0.3 mM *N,N,N’,N’*-tetramethyl-p-phenylenediamine (TMPD)] was added and oxygen consumption was detected. The auto-oxidation of the substrate was evaluated in parallel measurements in the absence of cells^51^.

### Confocal laser scanning microscopy analysis

Confocal laser scanning microscopy analysis were performed as previously described^13^. LoVo cells (8 × 10^3^/well) were seeded in 24-well plate containing microscope glass. After treatment, cells were fixed using 4% (v/v) paraformaldehyde solution for 20 min and then permeabilized with 0.1% (v/v) Triton X-100 in PBS for 10 min at RT. SIRT6 immunofluorescence detection was performed by using specific antibodies against Actin (1:1000) and SIRT6 (1:500). Alexa Fluor 488 (1:1000) or Alexa Fluor 633 (1:1000) were used as secondary antibodies. Zeiss LSM 700 confocal microscope with a plan apochromat X63 (NA1.4) oil immersion objective was utilized to perform microscopy analyses, as previously described^13^. The fluorescence intensity was estimated with ImageJ software 1.52n version and expressed as arbitrary fluorescence units (AFU).

### Statistical analysis

The data are represented as mean±standard deviation (SD). Statistical analysis was performed using One-way ANOVA followed by Bonferroni’s *post-hoc* tests. For IC_50_ determination, GraphPad Prism 6 was used to calculate statistical significance. *P* values <0.05 were considered to be statistically significant. All the experiments were performed in quadruplicates.

## Supplementary information

Supplementary information.
