# Supplementary material for: ROS-Mediated Apoptotic Cell Death of Human Colon Cancer LoVo Cells by Milk δ-Valerobetaine
Source: Sci Rep. 2020 Jun 2;10:8978. doi: 10.1038/s41598-020-65865-6 (PMC7265370; doi:10.1038/s41598-020-65865-6)
Supplement: Supplementary file 1 — Supplementary information. [file 41598_2020_65865_MOESM1_ESM.pdf]

ROS-Mediated Apoptotic Cell Death of Human Colon Cancer LoVo Cells by Milk δ-Valerobetaine

Nunzia D'Onofrio[a], Nunzio Antonio Cacciola[bc], Elisa Martino[a], Francesca Borrelli[d], Ferdinando Fiorino[d], Assunta Lombardi[e], Gianluca Neglia[b], Maria Luisa Balestrieri[a]*, Giuseppe Campanile[b]

[a]Department of Precision Medicine, University of Campania "L. Vanvitelli", 80138 Naples, Italy.

[b]Department of Veterinary Medicine and Animal Productions, University of Naples Federico II, 80137 Naples, Italy.

[c]Institute of Sustainable Plant Protection (IPSP), National Research Council (CNR), 80055 Naples, Italy.

[d] Department of Pharmacy, School of Medicine and Surgery, University of Naples Federico II, 80131 Naples, Italy.

[e]Department of Biology, University of Naples Federico II, 80126 Naples, Italy.

***Correspondence**: Maria Luisa Balestrieri, Department of Precision Medicine, University of Campania "L. Vanvitelli", via L. De Crecchio 7, 80138, Naples, Italy.

Tel.: +39 081 5665865

Fax: +39 081 5665863

Email: marialuisa.balestrieri@unicampania.it

ORCID: 0000-0001-6001-1789

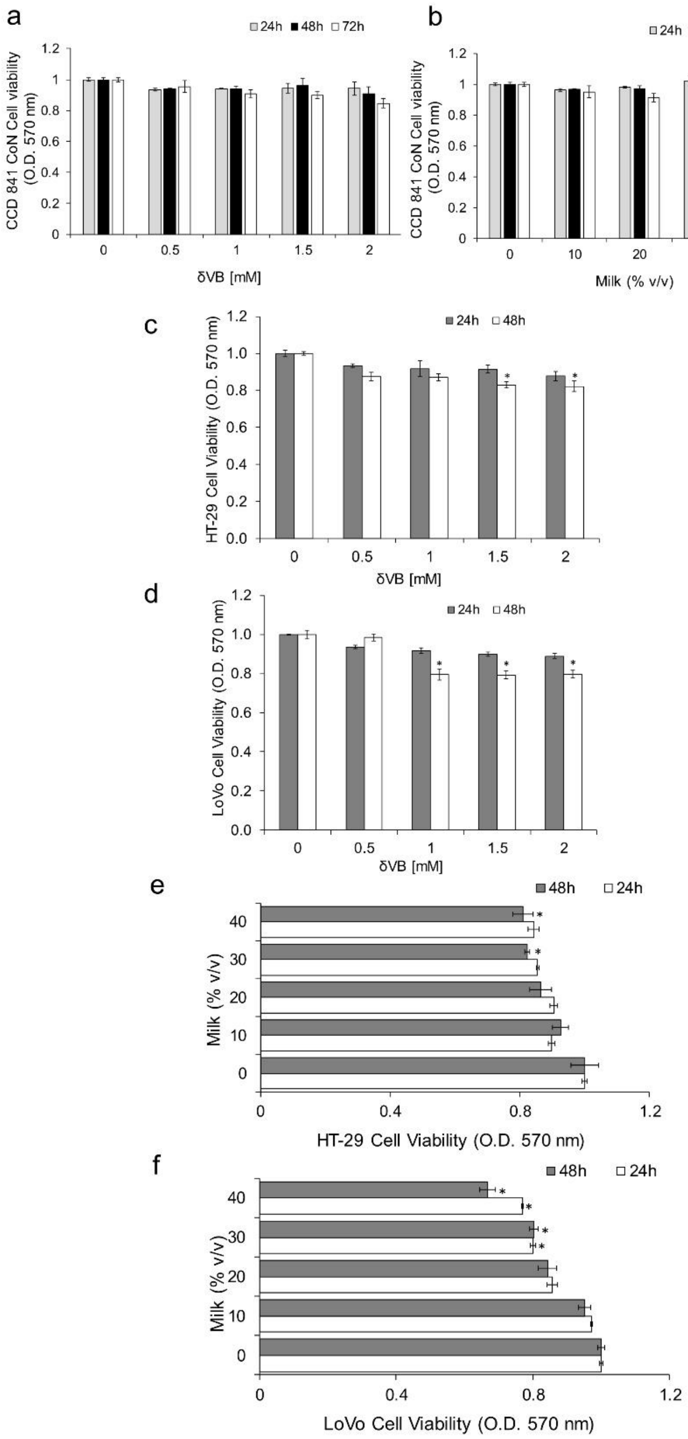

Figure. S1. Effects of milk-δVB on human non-malignant and colorectal adenocarcinoma cell viability. (**a, b**) CCD 841 CoN cells were treated with δVB (up to 2 mM) and milk (up to 40 % v/v) for 24, 48-72h. HT-29 and LoVo cells were treated with (**c, f**) increasing concentrations of δVB (up to 2 mM) and (**d, e**) milk (up to 40 % v/v) for 24-48 h. Control cells were grown in medium containing the same volume (% v/v) of HBSS-10 mM Hepes. Cell growth inhibition was assessed using MTT assay. Values represent the mean±SD of three independent experiments. $*P<0.05$ *vs* Ctr.

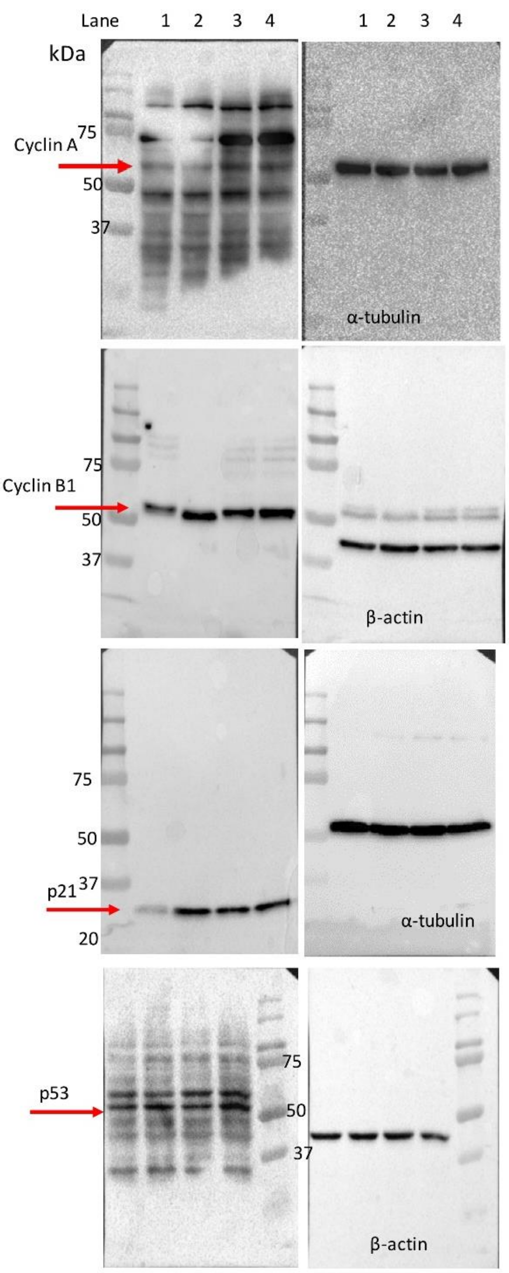

Figure. S2. LoVo cell cycle alteration induced by milk-δVB. The cropped blots are used in the main figure (Figure 2 c-f). Lane 1=Ctr; Lane 2=milk, Lane 3=δVB, Lane 4= milk+ δVB.

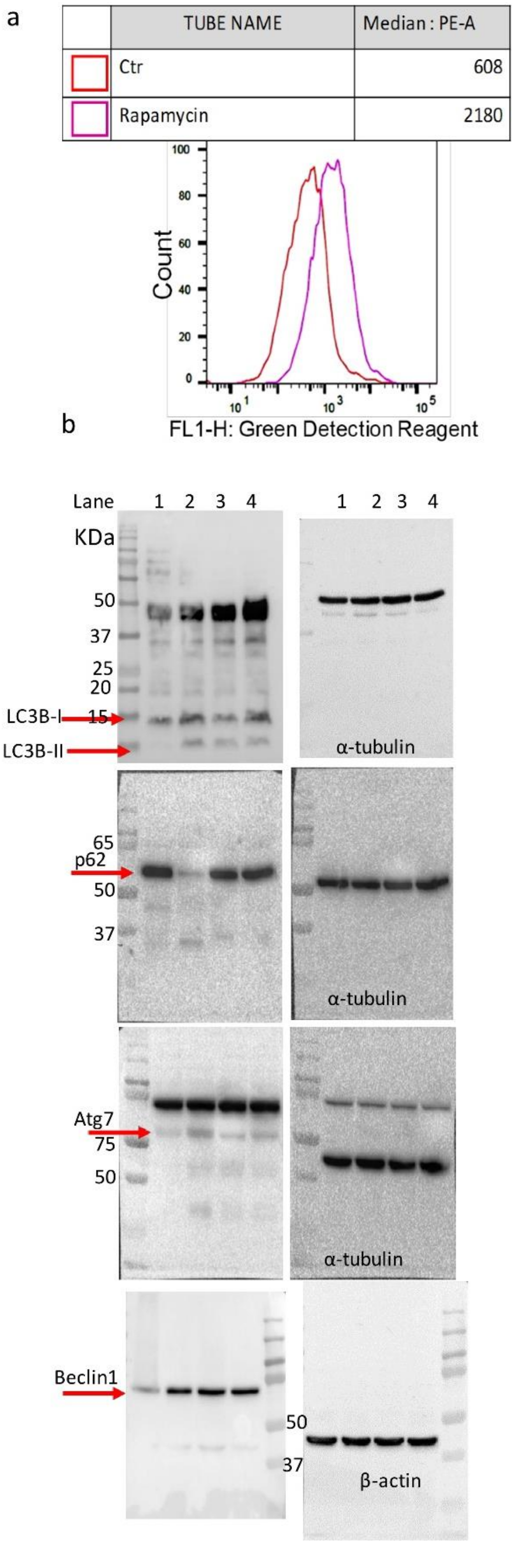

Figure. S3. Autophagy induced by milk-δVB. (a) Representative cytofluorimetric analysis of Green detection reagent performed in untreated LoVo cells (Ctr) and rapamycin-treated cell, as positive control of autophagic process. (b) The cropped blots are used in the main figure (Figure 3d-g). Lane 1=Ctr; Lane 2=milk, Lane 3=δVB, Lane 4= milk+ δVB.

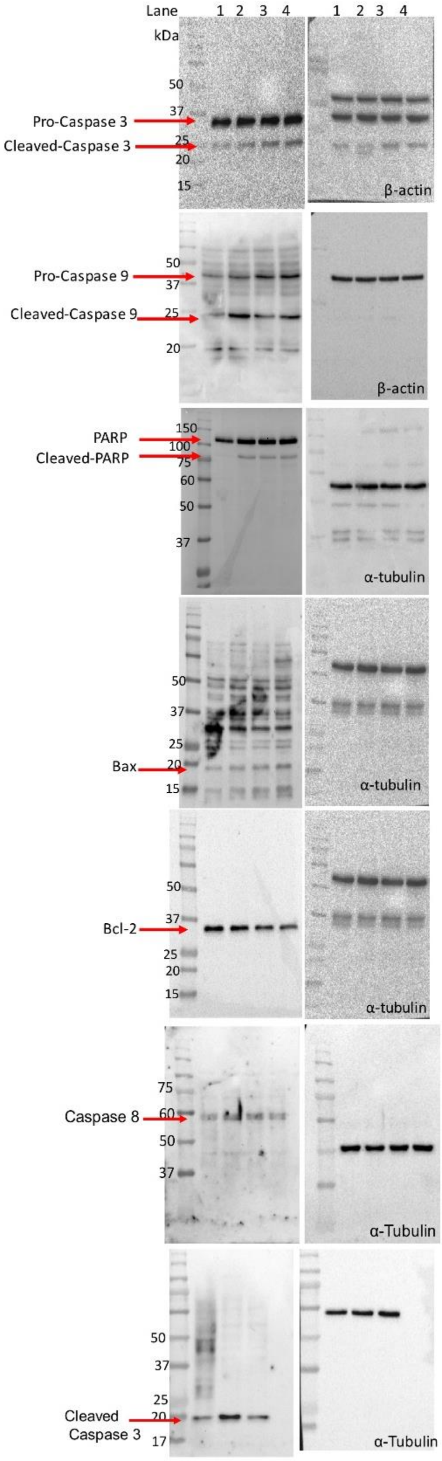

Figure. S4. Apoptotic mechanism. The cropped blots are used in the main figure (Figure 5c-h, n). Antibodies against Bax, Bcl-2 and SIRT6 (reported in the main Fig. 4) were blotted on the same filter and quantified by using the same loading control (α-tubulin). Lane 1=Ctr; Lane 2=milk, Lane 3=δVB, Lane 4= milk+ δVB. The cropped blots used in the main figure (Figure 5m). Lane 1=Ctr; Lane 2=milk+ δVB, Lane 2=Z-LEHD-FMK +milk+ δVB.

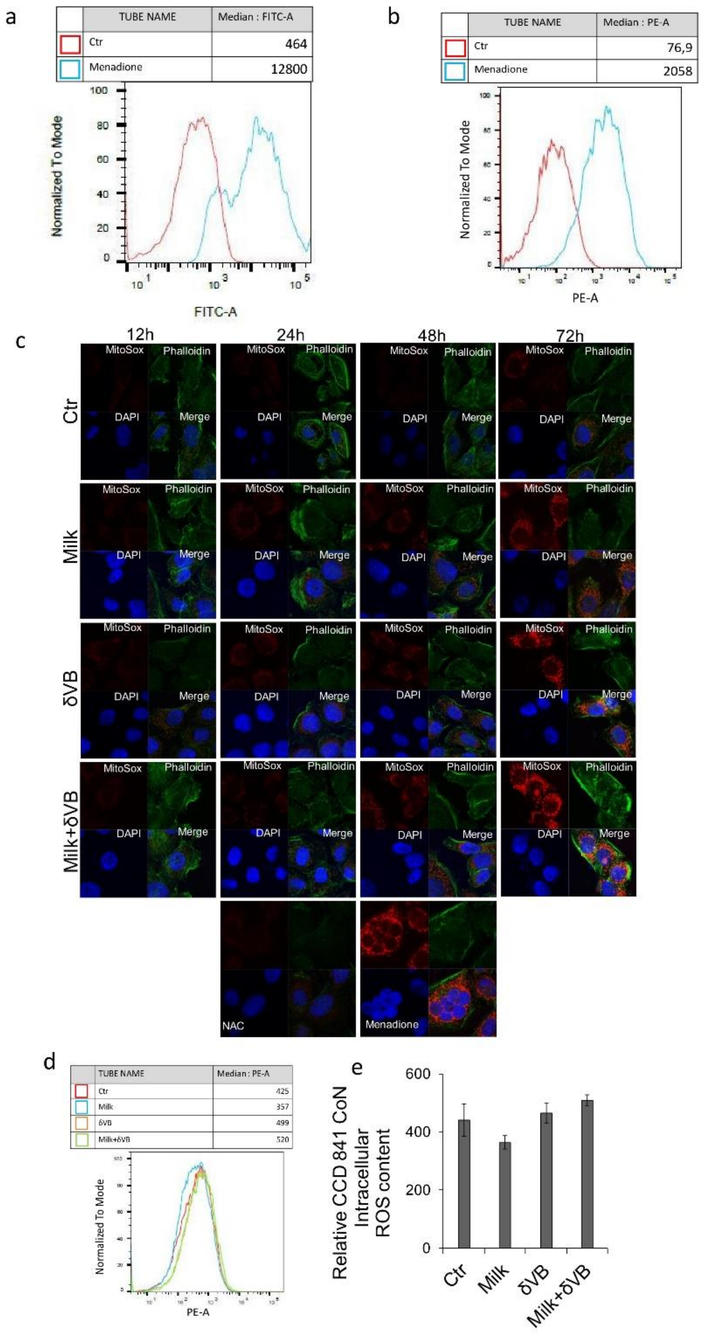

Figure. S5. Evaluation of redox homeostasis. Representative cytofluorimetric analysis of **(a)** DCFH-DA and (**b)** MitoSox assays performed in untreated LoVo cells (Ctr) and menadione-treated cells, as positive control of intracellular and mitochondrial ROS generation. **(c)** Representative confocal images peformed by 63X oil immersion objective of time-line (12, 24, 48, 72 h) mitochondrial ROS generation from milk (40% v/v), δVB (2 mM), milk+ δVB, or HBSS-10 mM Hepes (40% v/v) (Ctr) treated LoVo cells. Scale bars= 50 µm. **(d, e)** Representative FACS analysis and bar graph of DCFH-DA staining performed in CCD 841 CoN cells after 72 h of treatment with 40% v/v buffalo milk (milk), δVB (2 mM) and milk supplemented with δVB (milk+ δVB).

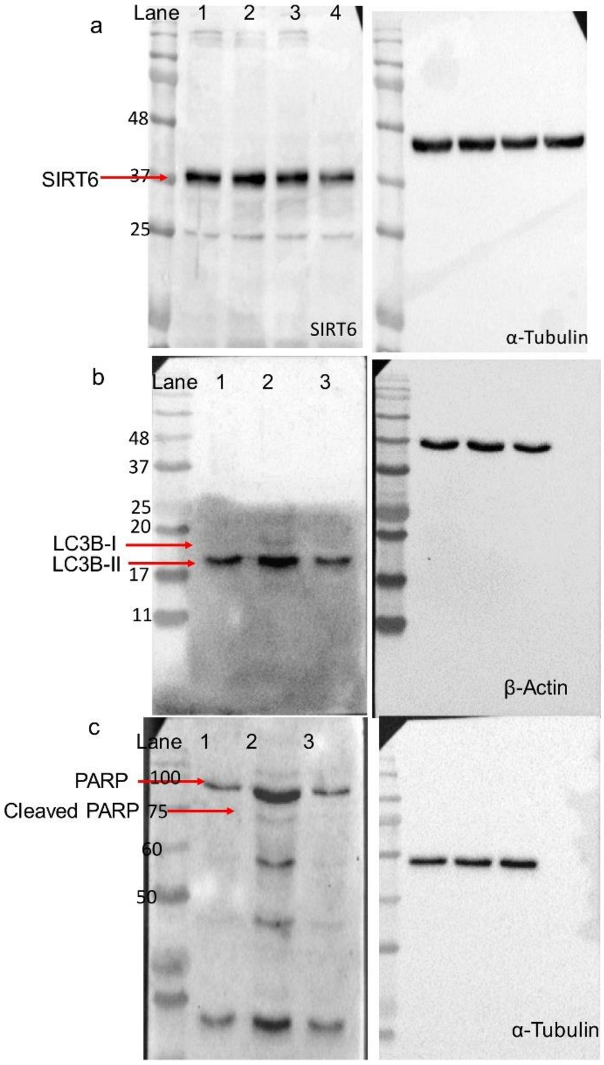

Figure. S6. The cropped blots are used in the main figure (Figure 7). (a) Lane 1=Ctr; Lane 2=milk, Lane 3=δVB, Lane 4= milk+ δVB. Lane 1= Ctr, Lane 2= Vehicle, Lane 3= Scramble, Lane 4= SIRT6-siRNA. (b, c) Lane 1= Ctr, Lane 2= milk+ δVB, Lane 3= SIRT6-siRNA+ milk+ δVB.
